# Necrotizing Pneumonia With Extensive Lobar Cavitation

**DOI:** 10.7759/cureus.56437

**Published:** 2024-03-19

**Authors:** Maxim Suleac, Socrates Naranjo, Malam Djassi, Isabel Lavadinho

**Affiliations:** 1 Internal Medicine Department, Unidade Local de Saúde do Norte Alentejano, Portalegre, PRT

**Keywords:** necrotizing pneumonia, tuberculosis, pleural effusion, dyspnea, cavitation

## Abstract

Pneumonia occupies one of the leading positions in morbidity and mortality worldwide. It is frequently categorized depending on the site of acquisition. Here, we present a case of a young woman who was admitted to the Emergency Department (ED) with cough, dyspnea, fever, and progressive worsening associated with palpitations and hypotension. An initial x-ray was followed by a computed tomography (CT) scan of the chest, which revealed signs of extensive left lung pneumonia with pleural effusion. Despite initial improvement after antibiotic treatment, the patient's condition declined. A repeat chest CT showed evidence of extensive lobar cavitations, leading to suspicion of tuberculosis.

## Introduction

Community-acquired pneumonia (CAP) can have different presentations, varying from a mild form characterized by fever and productive cough to a severe one manifested by respiratory distress and sepsis. It is believed to be the second most common cause of hospitalization and a common infectious cause of death [1]. When CAP is complicated by necrosis and abscesses, it is called necrotizing pneumonia (NP) [2]. This is a serious complication caused by particularly virulent bacteria. The most frequent agent that causes necrotizing pneumonia is pneumococcus [3-5]. Streptococcus pneumonia, serotype three, can cause severe necrosis and toxin production that destroys tissue even after eradication [3,6,7].

The heptavalent vaccine for pneumococcal vaccination was introduced in Portugal in 2001, but it was not part of the National Vaccination Program (PNV) initially. Prior to the introduction of the vaccine, the serotypes found in the seven-valent conjugate vaccine were responsible for about 60% of Invasive Pneumococcal Disease (IPD) in pediatric patients [8]. There is no exact data on vaccination coverage, but it is estimated that it was around 70% to 80% in 2007. Since 2009, pneumococcal vaccination has been included in the PNV.

## Case presentation

We present a clinical case of a 37-year-old female with a history of arterial hypertension, obesity, dyslipidemia, and tobacco use, with an updated PNV. The patient was admitted to the Emergency Department with symptoms of cough, dyspnea, and fever lasting for four days, with progressive worsening associated with palpitations and hypotension. Upon initial assessment, the patient was conscious, fully oriented, diaphoretic, and tachypneic with supplemental oxygen via nasal cannula at 2 L/min. Cardiac auscultation revealed no abnormalities. Pulmonary auscultation detected decreased vesicular murmur in the left hemithorax with rhonchi and crepitus. Vital signs were obtained with blood pressure 104/51 mmHg and heart rate 152-186 beats per minute. The Electrocardiogram (ECG) revealed signs of atrial fibrillation with 179 beats per minute, without ischemic alterations. Laboratory results showed leukocytosis, increased C-reactive protein, erythrocyte sedimentation rate, and procalcitonin (Table 1).

**Table 1:** Laboratory tests

Parameter	Normal	Patient
Leukocytos	4 - 6 x10^6^/µL	36.86 - 6 x10^6^/µL
C-reactive protein	0 - 0.5 mg/dL	40.5 mg/dL
Procalcitonin	0 - 0.5 ng/mL	3.32 ng/mL
Erythrocyte sedimentation rate	≤ 20 mm/hr	120 mm/hr

**Figure 1:**
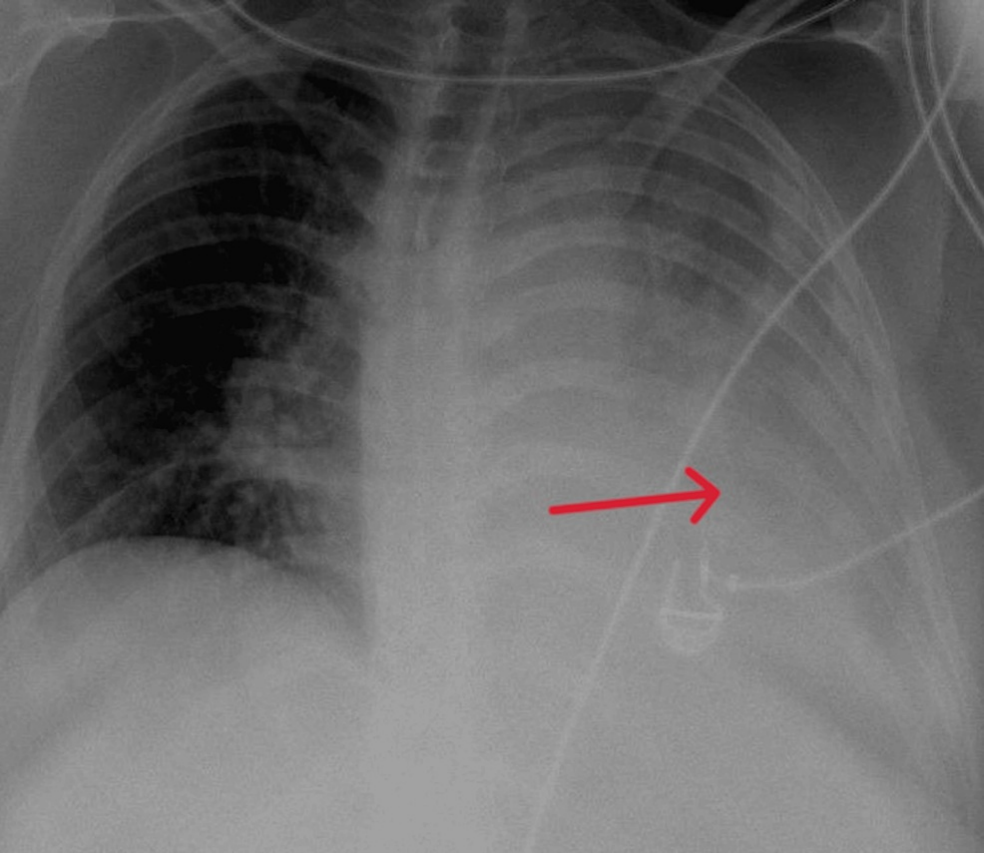
Extensive consolidation

**Figure 2:**
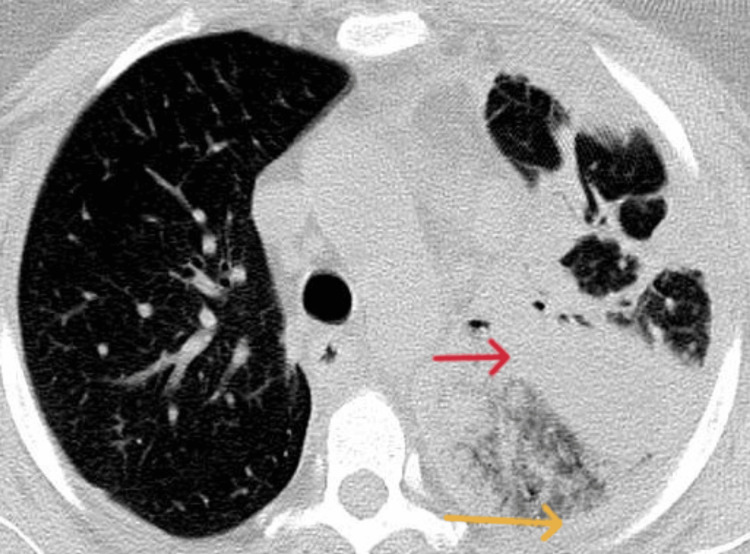
Pleural effusion (yellow arrow) with consolidation (red arrow)

**Figure 3:**
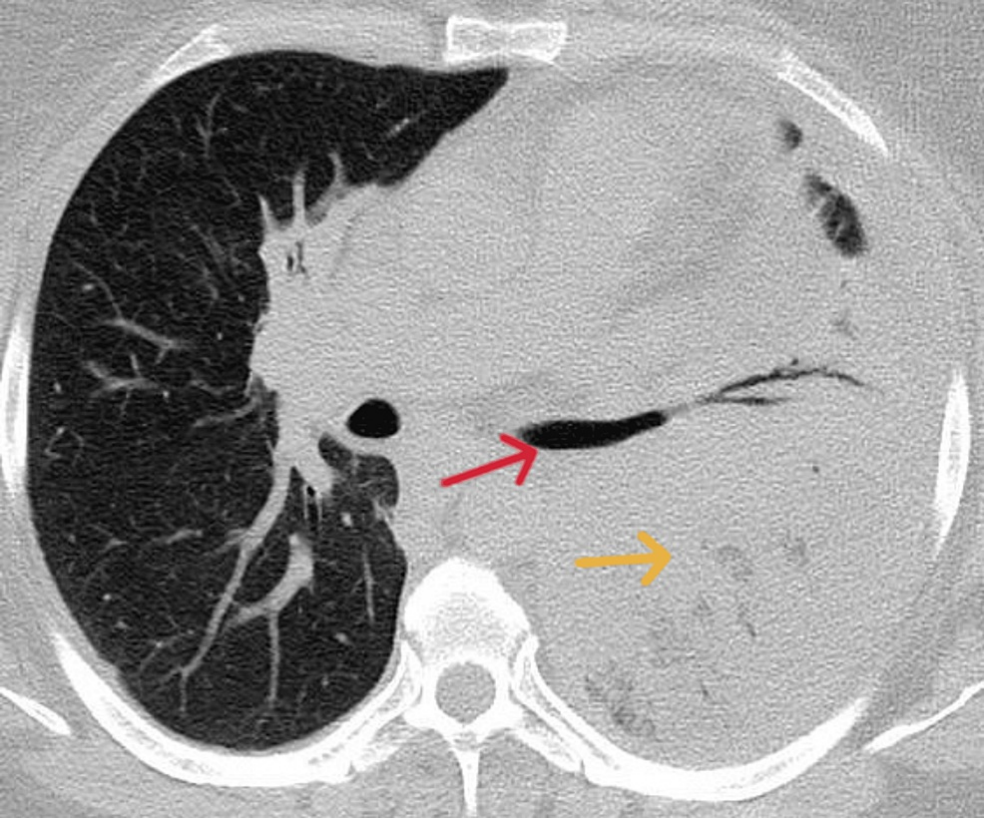
Extensive consolidation (yellow arrow) and air bronchogram (red arrow)

Test results for Legionella, pneumococcal antigen, and COVID-19 were negative. Blood gas analysis showed respiratory insufficiency type one. A chest x-ray (Figure 1) detected an extensive left lung consolidation, followed by the computed tomography (CT) scan revealed pneumonia affecting the entire left lung with air bronchogram, extensive consolidation, and pleural effusion, without the presence of bronchiectasis, cavitations, or signs of pulmonary emphysema (Figures 2, 3).

**Figure 4:**
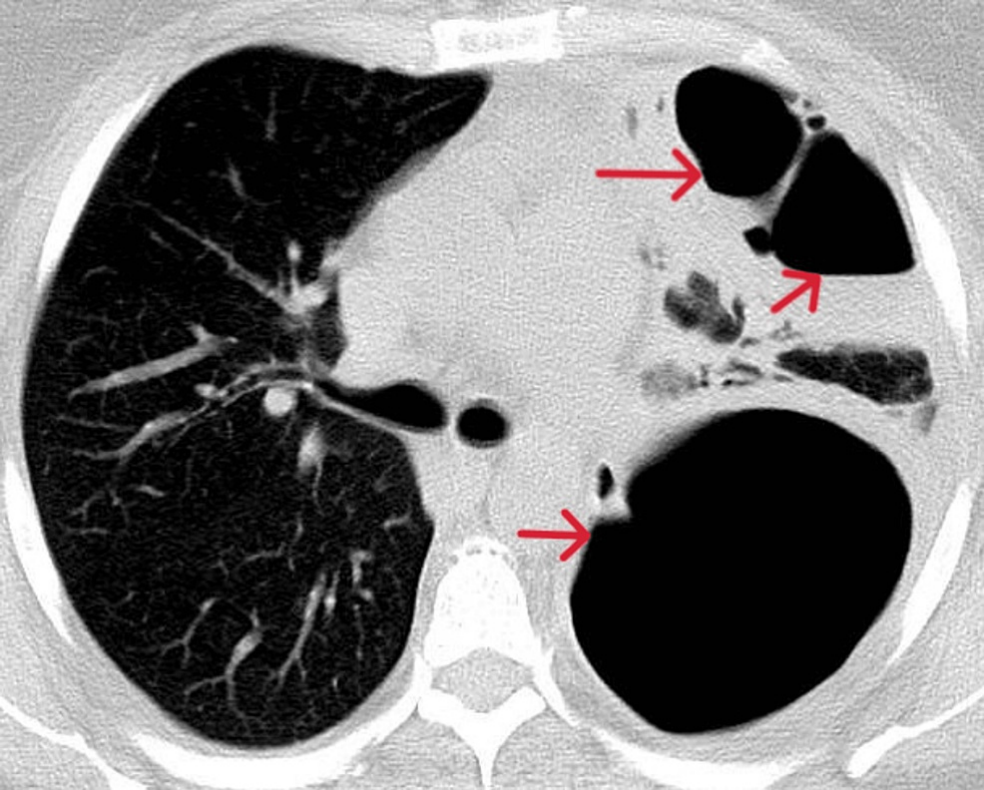
Cavitations (red arrows)

Blood cultures were collected, and empiric treatment with ceftriaxone and azithromycin was initiated. The patient has completed 10 days of ceftriaxone and five days of azithromycin treatment, which led to clinical and laboratory signs of improvement. Two days after finishing the antibiotic therapy, the peripheral oxygen saturation suddenly started to decrease without the patient complaining of dyspnea. A repeated CT scan of the chest (two-week interval) revealed extensive lobar cavitations with an area of consolidation and basal segmental bronchogram with associated mild adjacent pleural effusion, which may be compatible with tuberculosis (Figure 4).

**Figure 5:**
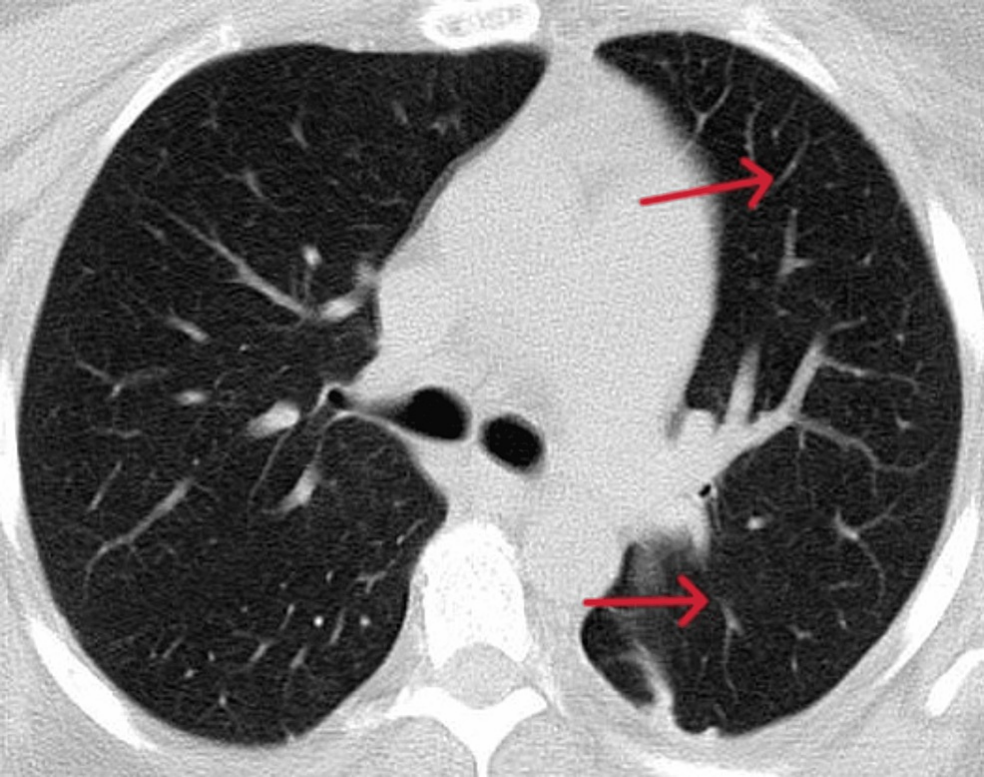
Recovery of the extensive pneumonia and complete resolution of pulmonary cavitations

Sputum was collected for exclusion/confirmation of tuberculosis [9]. The direct mycobacteriological study and culture tests were negative. Blood culture tests were also negative. Legionella and pneumococcal antigens were repeated, and the pneumococcal antigen was positive. Since the patient's condition worsened and taking into consideration a possible resistance to the previous antibiotic regimen, it was decided to continue treatment using broader-spectrum antibiotics, so treatment with piperacillin/tazobactam (21 days) and clindamycin (13 days) was initiated. To further investigate, a bronchofibroscopy with bronchoalveolar lavage was performed. The sample was sent for mycology, bacteriology, mycobacteriology, and cytology study. All the collected samples tested negative. The patient has been responding well to the proposed therapy, with good clinical evolution and decreased inflammatory parameters without the need for supplemental oxygen. The control CT scan showed the resolution of the disease process (Figure 5). Finally, the patient was discharged from the hospital with subsequent follow-up at the clinic.

## Discussion

NP is generally regarded as a rare complication of pneumococcal infection in adults [10], characterized by the destruction of lung parenchyma resulting in multiple cavitations, often accompanied by empyema. NP is diagnosed through serial radiography and thoracic CT scans. Surgical resection may be warranted in cases of sepsis, persistent fever, empyema, or failure to respond to antibiotic therapy [11,12].

Type 3 pneumococcus was more frequently isolated from patients with necrosis than those without necrosis, although ten other serotypes were also implicated [11,13]. In Portugal, all children up to 12 months of age receive three doses of the Pn13 vaccine. The vaccine is only re-administered to the population over 65 years of age or to special risk groups (e.g., immunocompromised individuals). Our patient had several risk factors for severe pneumonia but did not fall within the guidelines for vaccination in adulthood. Some authors have demonstrated that the most common pneumococcal serotypes in adulthood are also included in the Pn13 vaccine administered during childhood, raising questions about the efficacy/durability of immunity acquired in infancy [14-16].

Moreover, the impact of antibiotic resistance patterns in Streptococcus pneumoniae strains causing NP is noteworthy [16]. Antibiotic resistance can significantly influence treatment outcomes and may necessitate adjustments in therapeutic strategies.

Exploring the host immune response to pneumococcal infection in the context of NP is crucial [16,17]. Factors such as host immune status, inflammatory cytokine profiles, and genetic predispositions may influence disease severity and outcomes. Recent advancements in diagnostic techniques for detecting pneumococcal pneumonia, including molecular assays and biomarker-based approaches, offer improved sensitivity and specificity compared to conventional culture-based methods [18]. These advancements can facilitate earlier and more accurate diagnosing, improving patient management and outcomes. Notably, the initial antigen test for pneumococcus yielded a negative result. The literature reports a sensitivity of approximately 74% when utilizing modern immunochromatographic tests, significantly surpassing the sensitivity of culture tests (which typically fall below 30%) [19]. However, it is important to acknowledge that despite these advancements, there remains room for improvement, as these tests are not yet perfect.

## Conclusions

The broader public health implications of NP caused by Streptococcus pneumoniae cannot be overlooked. NP caused by Streptococcus pneumoniae is a rare but serious complication characterized by destructive lung parenchyma changes often accompanied by empyema. Diagnosis relies on radiographic imaging and clinical evaluation, with surgical intervention being necessary in severe cases. The prognosis and clinical outcomes were good in the presented clinical case. The suggested treatment was appropriate, but there is still scope for improvement, especially in the currently used pneumonia testing. Early detection and initiation of treatment lead to better patient outcomes with further scope for improvement and evidence-based research. Our case study highlights the importance of considering pneumococcal vaccination strategies, particularly in adults with underlying risk factors, to mitigate the risk of severe pneumococcal infections. Public health initiatives to increase pneumococcal vaccination coverage are essential for preventing the cases of NP caused by Streptococcus pneumoniae. Further research is warranted to elucidate the complex pathogenesis of this condition and to develop more effective preventive and therapeutic strategies.
